# Honokiol suppresses formyl peptide-induced human neutrophil activation by blocking formyl peptide receptor 1

**DOI:** 10.1038/s41598-017-07131-w

**Published:** 2017-07-27

**Authors:** Fu-Chao Liu, Huang-Ping Yu, Yu-Ting Syu, Jia-You Fang, Chwan-Fwu Lin, Shih-Hsin Chang, Yen-Tung Lee, Tsong-Long Hwang

**Affiliations:** 1 Department of Anesthesiology, Chang Gung Memorial Hospital, Taoyuan, 333 Taiwan; 2 School of Medicine, College of Medicine, Chang Gung University, Taoyuan, 333 Taiwan; 3 Graduate Institute of Natural Products, College of Medicine, Chang Gung University, Taoyuan, 333 Taiwan; 4 Chinese Herbal Medicine Research Team, Healthy Aging Research Center, Chang Gung University, Taoyuan, 333 Taiwan; 5 Department of Cosmetic Science, College of Human Ecology, Chang Gung University of Science and Technology, Taoyuan, 333 Taiwan; 6 Research Center for Chinese Herbal Medicine, Research Center for Food and Cosmetic Safety, and Graduate Institute of Health Industry Technology, College of Human Ecology, Chang Gung University of Science and Technology, Taoyuan, 333 Taiwan; 7 Division of Natural Products, Graduate Institute of Biomedical Sciences, College of Medicine, Chang Gung University, Taoyuan, 333 Taiwan

## Abstract

Formyl peptide receptor 1 (FPR1) mediates bacterial and mitochondrial *N*-formyl peptides-induced neutrophil activation. Therefore, FPR1 is an important therapeutic target for drugs to treat septic or sterile inflammatory diseases. Honokiol, a major bioactive compound of Magnoliaceae plants, possesses several anti-inflammatory activities. Here, we show that honokiol exhibits an inhibitory effect on FPR1 binding in human neutrophils. Honokiol inhibited superoxide anion generation, reactive oxygen species formation, and elastase release in bacterial or mitochondrial *N*-formyl peptides (FPR1 agonists)-activated human neutrophils. Adhesion of FPR1-induced human neutrophils to cerebral endothelial cells was also reduced by honokiol. The receptor-binding results revealed that honokiol repressed FPR1-specific ligand *N*-formyl-Nle-Leu-Phe-Nle-Tyr-Lys-fluorescein binding to FPR1 in human neutrophils, neutrophil-like THP-1 cells, and hFPR1**-**transfected HEK293 cells. However, honokiol did not inhibit FPR2-specific ligand binding to FPR2 in human neutrophils. Furthermore, honokiol inhibited FPR1 agonist-induced calcium mobilization as well as phosphorylation of p38 MAPK, ERK, and JNK in human neutrophils. In conclusion, our data demonstrate that honokiol may have therapeutic potential for treating FPR1-mediated inflammatory diseases.

## Introduction

Inflammation is a major feature of the host response against external pathogen invasion or internal pathologic progression. Immune responses are complex and essential, affecting organ function and the survival of an individual. Leukocyte activation and infiltration are expressions of the inflammatory and immune responses^1, 2^. About 40%-75% of circulating human leukocytes are neutrophils that not only serve as phagocytes but also generate inflammatory mediators to produce inflammatory stimuli^3, 4^. However, toxic reactive oxygen species (ROS) and proteolytic enzymes released by activated neutrophils can destroy the surrounding tissue^1, 5^. Therefore, the attenuation of neutrophil activation is a critical strategy to decrease the inflammatory response and thus alleviate inflammatory diseases^4, 6–8^.

Honokiol, a major bioactive compound derived from the Magnoliaceae plants, has several pharmacological effects including anticancer, antimicrobial, antioxidant, anti-inflammatory and organ-protective properties^9–13^. Honokiol can also inhibit neutrophil infiltration, suppress ROS generation and protect against heart and brain ischemia/reperfusion injury^14, 15^. Liu *et al*. has reported that honokiol attenuated rat traumatic spinal cord injury through downregulation of Kruppel-like factor 4 expression and suppression of the inflammatory response^16^. In addition, honokiol was found to be a natural inhibitor of nuclear (NF)-κB and to inhibit NF-κB-mediated gene expression, including the expression of tumor necrosis factor (TNF)-α, intercellular adhesion molecule-1, monocyte chemotactic protein-1 and matrix metalloproteinase-9 in various cell types^17^. Our previous study showed that honokiol attenuates vascular cell adhesion molecule-1 expression and neutrophil adhesion in TNF-α-induced cerebral endothelial cells (ECs)^18^. Neutrophils have significant roles in various inflammatory disorders and autoimmune diseases^4, 19^. Honokiol displays potent anti-inflammatory activity to reduce tissue damage^11, 14–16, 20^. However, the pharmacological effects and molecular target of honokiol in human neutrophils remain unclear.

Formyl peptide receptors (FPRs) are pattern recognition receptors that recognize exogenous pathogen-associated or endogenous damage-associated molecular patterns^8^. Previous researches have shown that FPR1 mediates neutrophil activation during inflammation^21, 22^. FPR1 can attract neutrophils to inflammatory site and mediate intracellular signal transduction in immune responses^23, 24^. FPR1 activation leads to either infectious or sterile inflammation by recognizing bacterial or mitochondrial *N*-formyl peptides^25, 26^. FPR1 antagonists have important anti-inflammatory effects based on *in vitro* and *in vivo* studies^8^. Therefore, inhibition of FPR1 may represent a novel therapeutic target for sterile or septic inflammatory diseases^27^. In this study, we sought to elucidate the exact mechanism of honokiol-associated anti-inflammatory activity in human neutrophils. The present results showed that honokiol inhibits *N*-formyl peptide-induced superoxide anion generation, elastase release, and cell adhesion in human neutrophils.

## Results

### Honokiol inhibits superoxide anion generation, elastase release, and ROS formation in *N*-formyl peptides-activated human neutrophils

**Figure 1:**
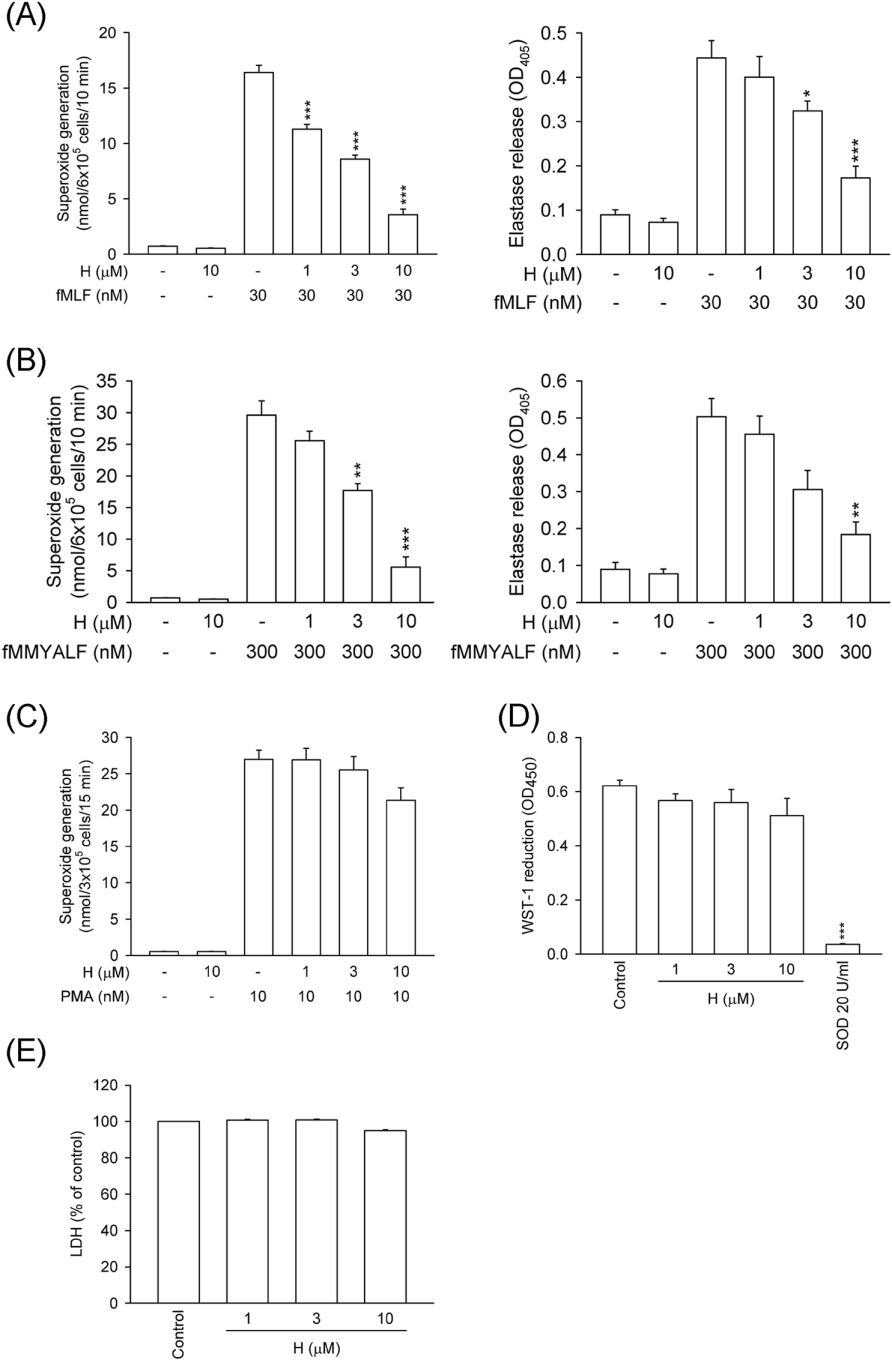
Honokiol inhibits superoxide anion generation and elastase release in FPR1-activated human neutrophils. Human neutrophils were incubated with 0.1% DMSO (as control) or honokiol (H; 1, 3, and 10 μM) for 5 min, and then activated by (**A**) fMLF (30 nM), (**B**) fMMYALF (300 nM), or (**C**) PMA (10 nM) for another 10 min. When fMLF and fMMYALF were used as activators, cells were primed by pre-incubation for 3 min with cytochalasin B (CB). Superoxide anion production was measured by ferricytochrome *c* reduction and elastase release was measured spectrophotometrically at 405 nm. (**D**) Xanthine oxidase was incubated with 0.1% DMSO (as control) or honokiol for 3 min, and then xanthine (0.1 mM) was added for 10 min. Reduction of WST-1 by cell-free xanthine/xanthine oxidase was measured spectrophotometrically at 450 nm. Superoxide dismutase (SOD; 20 U/ml) was used as a positive control. (**E**) Human neutrophils were incubated with 0.1% DMSO (as control) or honokiol for 15 min. Cytotoxicity was measured by assessing LDH release in cell-free medium. Total LDH release was determined by lysing cells with 0.1% Triton X-100 for 30 min at 37 °C. All data are expressed as the mean ± S.E.M. (*n* = 3–5). *P < 0.05, **P < 0.01, ***P < 0.001 compared with (**A**) fMLF, (**B**) fMMYALF, or (**D**) control (0.1% DMSO).

Superoxide anion generation, elastase release, and ROS formation are important indicators of respiratory burst and degranulation in activated human neutrophils. Honokiol (1, 3, and 10 μM) had dose-dependent inhibitory effects on superoxide anion generation and elastase release in human neutrophils activated by a bacterial FPR1 activator, *N*-formyl-Met-Leu-Phe (fMLF), with IC_50_ values of 3.31 ± 0.52 and 4.16 ± 0.32 μM, respectively (Fig. 1A). Moreover, honokiol also inhibited human mitochondrial *N*-formyl peptide, *N*-formyl-Met-Met-Tyr-Ala-Leu-Phe (fMMYALF)^28^, induced human neutrophil superoxide anion generation and elastase release with IC_50_ values of 4.01 ± 0.59 and 3.75 ± 0.81 μM, respectively (Fig. 1B). Honokiol (10 μM) did not alter superoxide anion generation and elastase release in resting human neutrophils.

Honokiol (1, 3, and 10 μM) did not show significant inhibitory effect on superoxide anion generation in phorbol 12-myristate 13-acetate (PMA, a protein kinase c activator)-induced human neutrophils (Fig. 1C). The superoxide anion-scavenging ability of honokiol was evaluated using a cell-free xanthine/xanthine oxidase system. Our data showed that honokiol (1, 3, and 10 μM) had no significant superoxide anion-scavenging effect. Superoxide dismutase (SOD, 20 U/ml) was used as a positive control (Fig. 1D). A cytotoxicity study was performed to determine whether the inhibitory effects of honokiol are mediated via cytotoxic effect. Honokiol (1, 3, and 10 μM) did not cause lactate dehydrogenase (LDH) release (Fig. 1E). These results suggested that its anti-inflammatory effects on neutrophil respiratory burst and degranulation were not due to superoxide anion scavenging or cytotoxicity.

**Figure 2:**
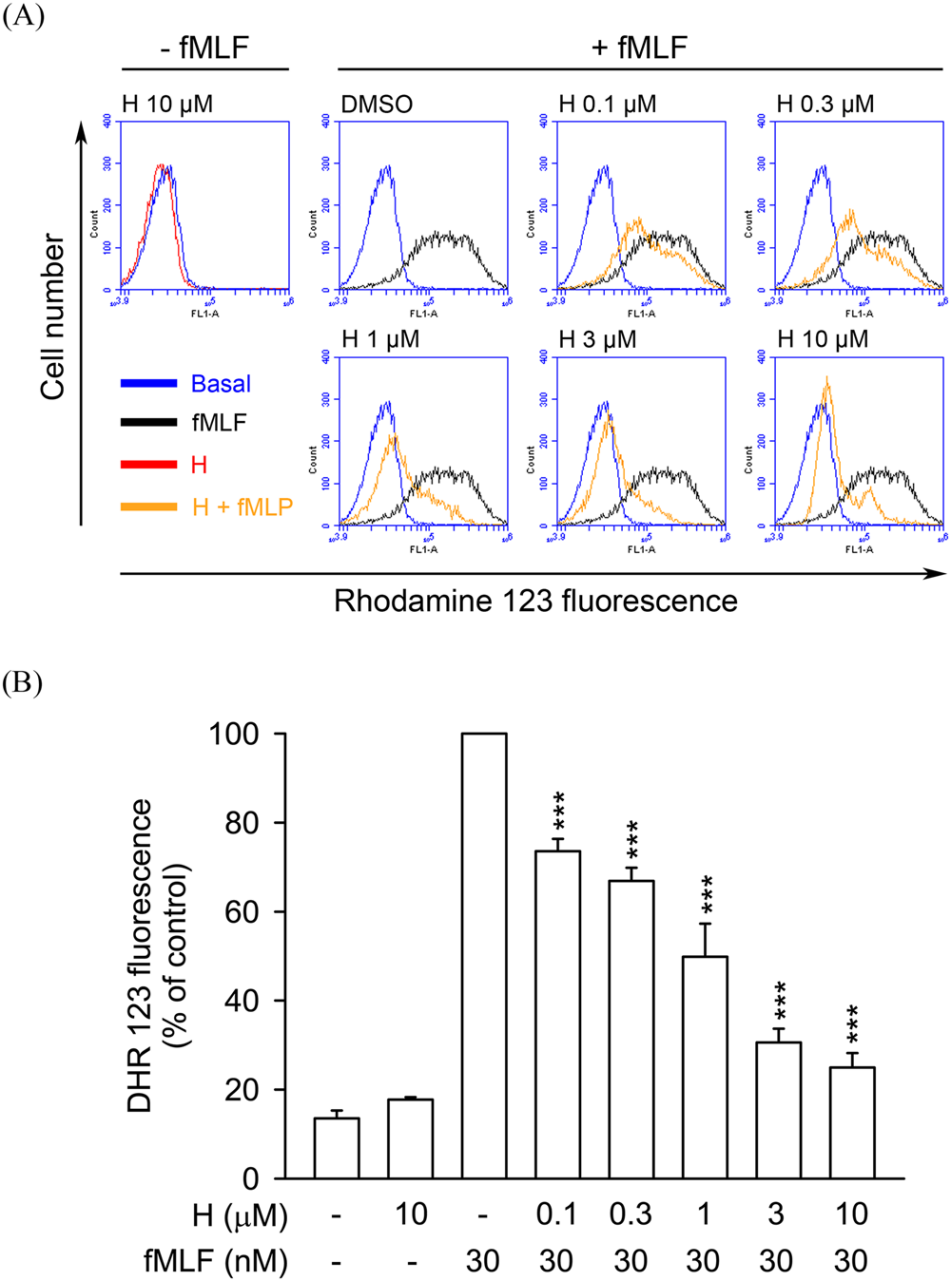
Honokiol inhibits ROS formation in fMLF-stimulated human neutrophils. Human neutrophils were labelled with DHR 123 (2 μM) and incubated with 0.1% DMSO (as control) or honokiol (H; 0.1–10 μM) for 5 min. Cells were activated using fMLF (30 nM) in the presence of CB (0.5 μg/ml) for a further 5 min. (**A**) Representative histograms showing typical fluorescent-activated cell sorting profiles for rhodamine 123 in unstimulated cells and in cells stimulated with fMLF in the absence or presence of honokiol at the concentration indicated. (**B**) Mean fluorescence intensity is shown as the mean ± S.E.M. (*n* = 5). ***P < 0.001 compared with fMLF alone.

The superoxide anion formed by NADPH oxidase in neutrophils can be converted to various ROS, leading to extensive tissue damage. Flow cytometric analysis showed that ROS formation in fMLF-stimulated human neutrophils was strongly inhibited by honokiol (0.1–10 μM) in a dose-dependent manner with an IC_50_ value of 1.28 ± 0.39 μM (Fig. 2).

In addition, human neutrophils were stimulated with other chemoattractants, including leukotriene B4 (LTB4), interleukin-8 (IL-8), and platelet activating factor (PAF), to induce elastase release. Honokiol, at low concentrations (1 and 3 μM), did not show significant inhibitory effect on these chemoattractants-induced elastase release (Supplementary Fig. S1). The IC_50_ values of honokiol on elastase release induced by LTB4, IL-8, and PAF were 6.51 ± 1.43, 6.86 ± 1.38, and >10 μM, respectively. These results suggest that honokiol has more sensitive inhibitory effects on FPR1 agonist-activated human neutrophils.

### Honokiol decreases FPR1-specific ligand binding to FPR1 in human neutrophils, neutrophil-like THP-1 cells, and FPR1-transfected HEK-293 cells

**Figure 3:**
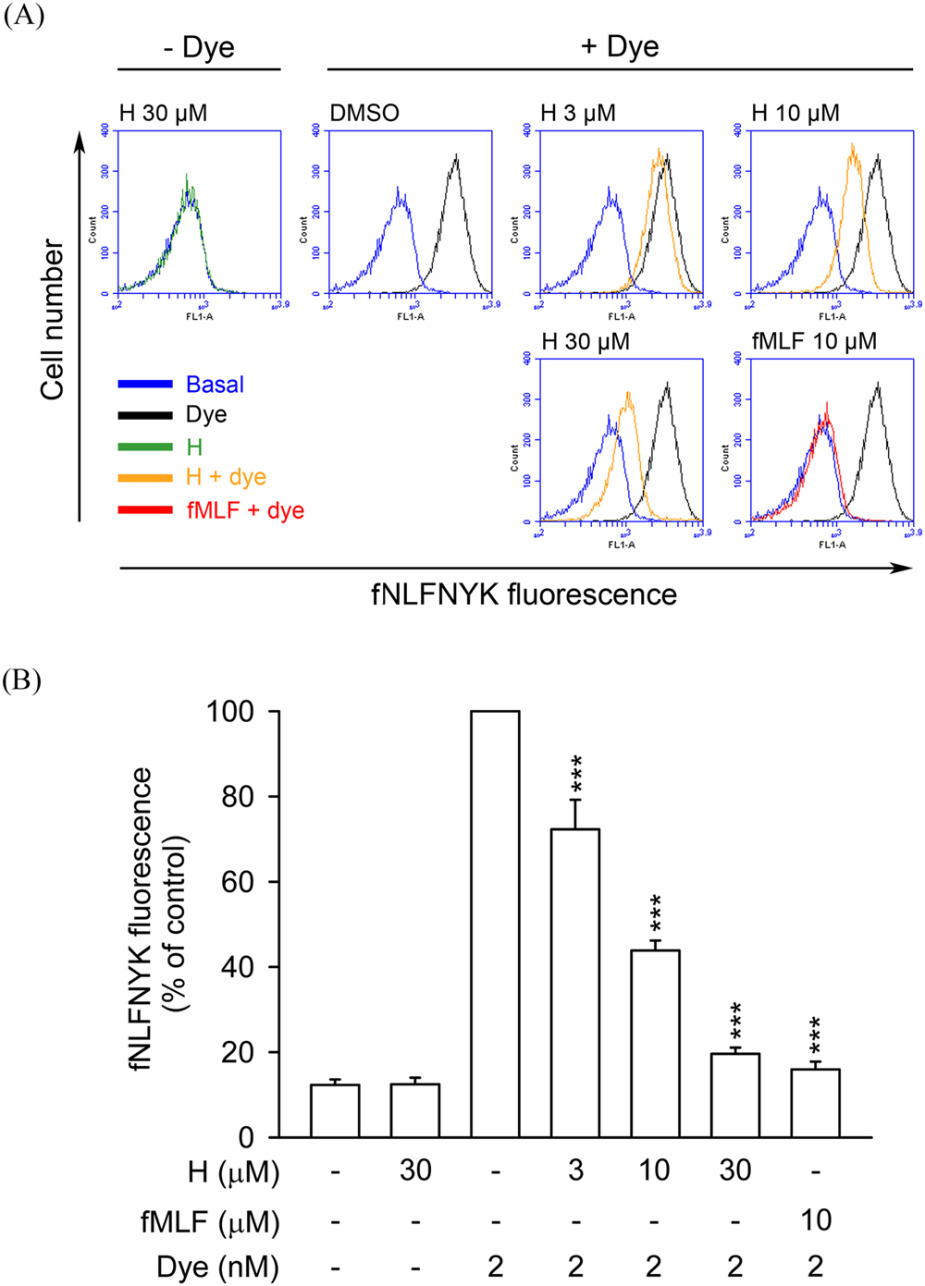
Honokiol blocks fNLFNYK binding to FPR1 in human neutrophils. Human neutrophils were incubated with 0.1% DMSO (as control), honokiol (H; 3, 10, and 30 μM), or fMLF (10 μM) for 10 min and then labelled with FPR1-specific fluorescent ligand fNLFNYK (2 nM) for 20 min. (**A**) Representative histograms showing typical fluorescence in the absence or presence of fNLFNYK with honokiol or fMLF. (**B**) Mean fluorescence intensity is shown as the mean ± S.E.M. (*n* = 4). **P < 0.01; ***P < 0.001 compared with fluorescent dye alone.

To examine whether honokiol has a binding affinity on FPR1, the receptor binding effect of *N*-formyl-Nle-Leu-Phe-Nle-Tyr-Lys-fluorescein (fNLFNYK), an FPR1-specific fluorescent ligand, on the surface of neutrophils was evaluated using flow cytometry^29^. The results showed that fMLF (10 μM) almost completely inhibited the binding of fNLFNYK (2 nM) on neutrophils. Compared with the control group, honokiol (3, 10, and 30 μM) significantly and dose-dependently inhibited the binding of fNLFNYK to FPR1 in human neutrophils with an IC_50_ value of 7.44 ± 0.97 μM (Fig. 3).

**Figure 4:**
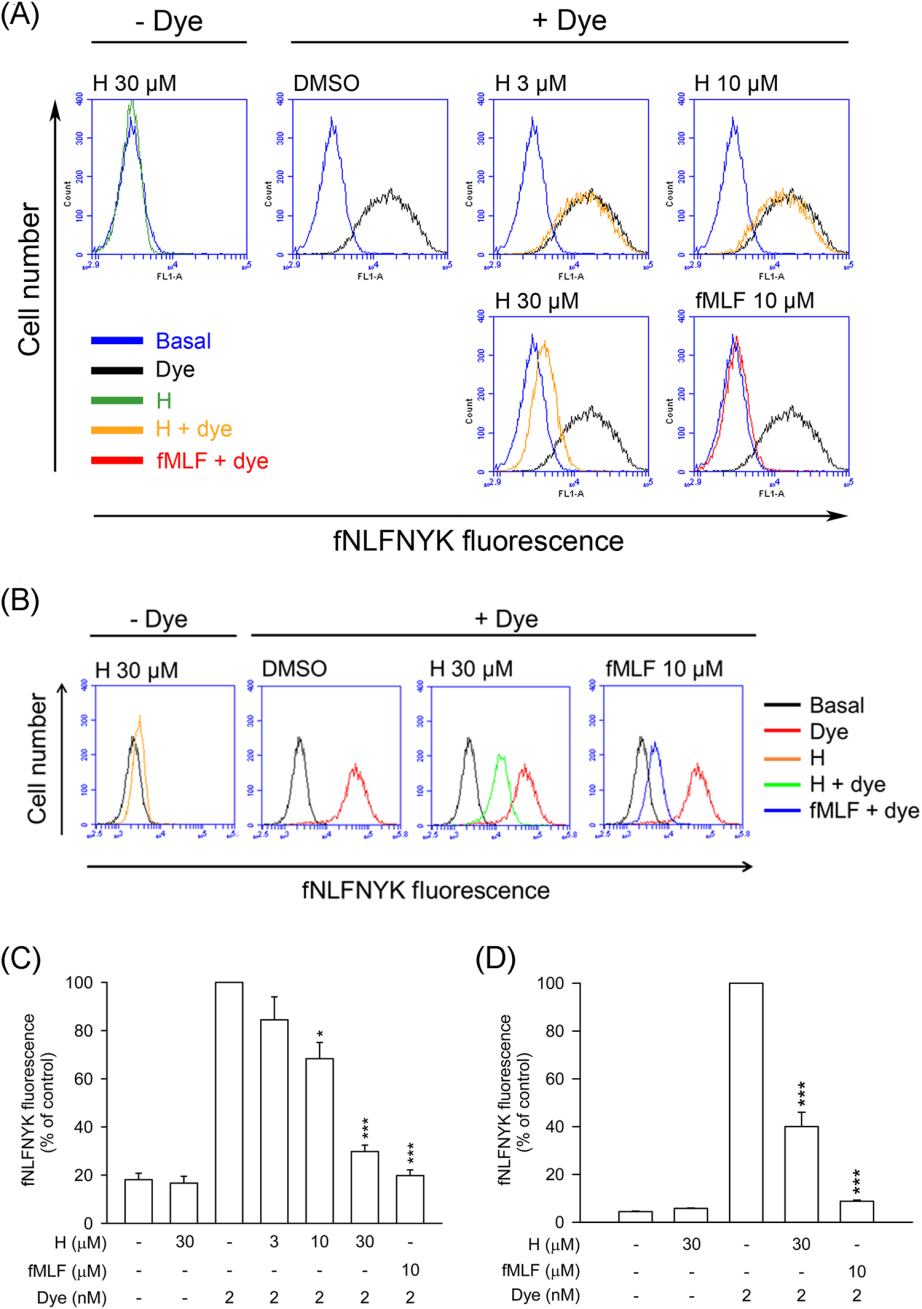
Honokiol blocks fNLFNYK binding to FPR1 in FPR1-expressed neutrophil-like cells. (**A**) Dibutyryl cAMP-differentiated THP-1 or (**B**) hFPR1-transfected HEK-293 cells were incubated with 0.1% DMSO (as control), honokiol (H; 3, 10, or 30 μM), or fMLF (10 μM) for 10 min and then labelled with fNLFNYK (2 nM) for 20 min. (**A**,**B**) Representative histograms showing typical fluorescence in the absence or presence of fNLFNYK with honokiol or fMLF. (**C** and **D**) Mean fluorescence intensities of (**A** and **B**) are shown as the mean ± S.E.M. (*n* = 3). *P < 0.05; **P < 0.01; ***P < 0.001 compared with fluorescent dye alone.

**Figure 5:**
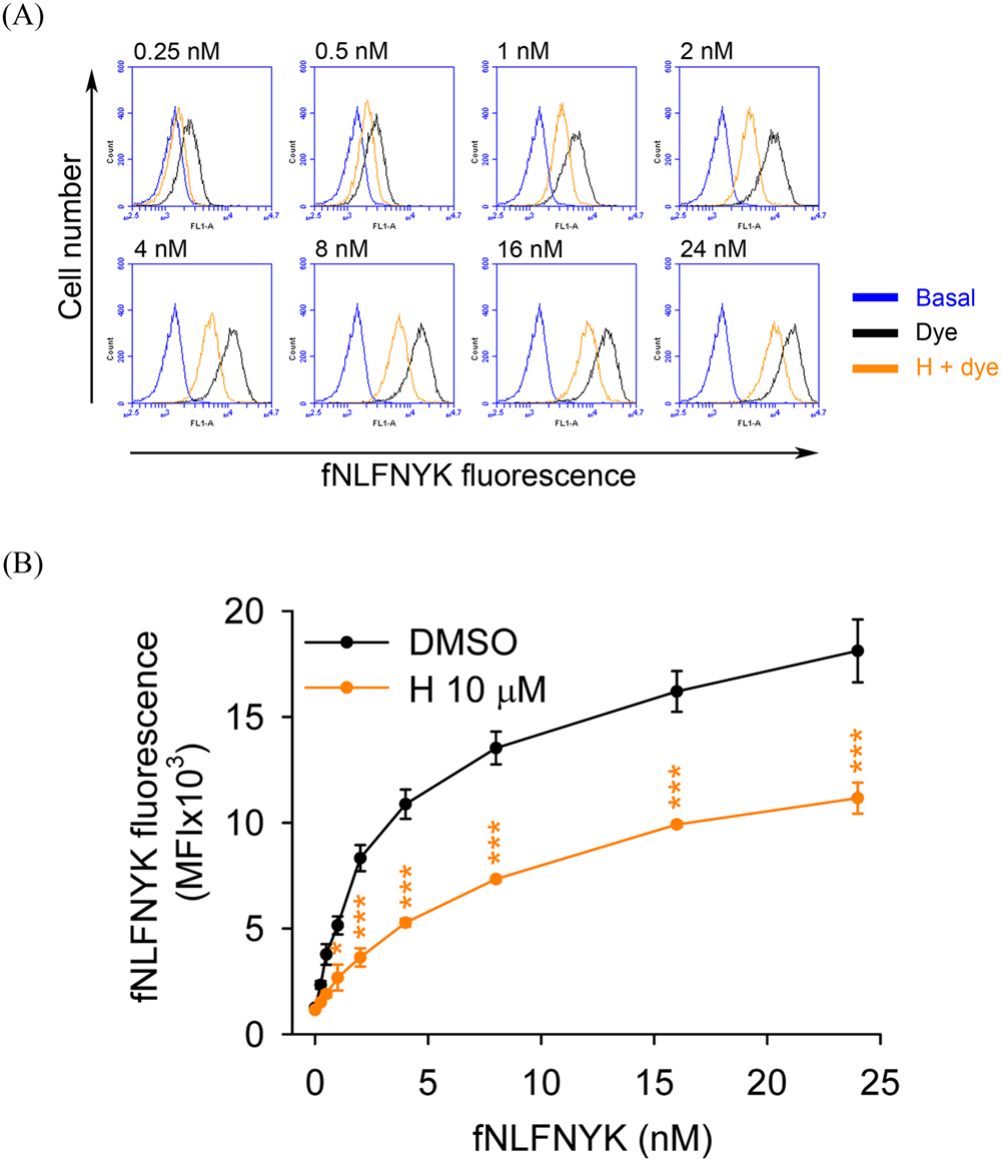
Inhibitory effect of honokiol on dose–response curve of fNLFNYK in human neutrophils. Human neutrophils were incubated with 0.1% DMSO (as control) or honokiol (H; 10 μM) for 10 min before labelling with different concentrations of fNLFNYK (0.25–24 nM) for 20 min. (**A**) Representative histograms showing typical fluorescence of fNLFNYK in the absence or presence of honokiol. (**B**) Mean fluorescence intensity is shown as the mean ± S.E.M. (*n* = 5). *P < 0.05; **P < 0.01 compared with corresponding data.

To confirm that honokiol has FPR1 binding affinity, the receptor-binding assay was performed in dibutyryl cAMP-differentiated THP-1 and hFPR1-transfected HEK-293 cells. Similar results showed that honokiol significantly suppressed the binding of fNLFNYK to FPR1 in both FPR1-expressed neutrophil-like cells (Fig. 4). These results again support the hypothesis that honokiol acts as a FPR1 inhibitor in human neutrophils. In addition, the binding capability of honokiol on the dose–response curve of fNLFNYK (0.25–24 nM) was examined. We found that the binding effects of high concentrations of fNLFNYK were also replaced by honokiol (Fig. 5), suggesting that honokiol binds to FPR1 in a high-affinity manner.

### Neither FPR1 mRNA expression nor FPR2 binding activity is altered by honokiol

**Figure 6:**
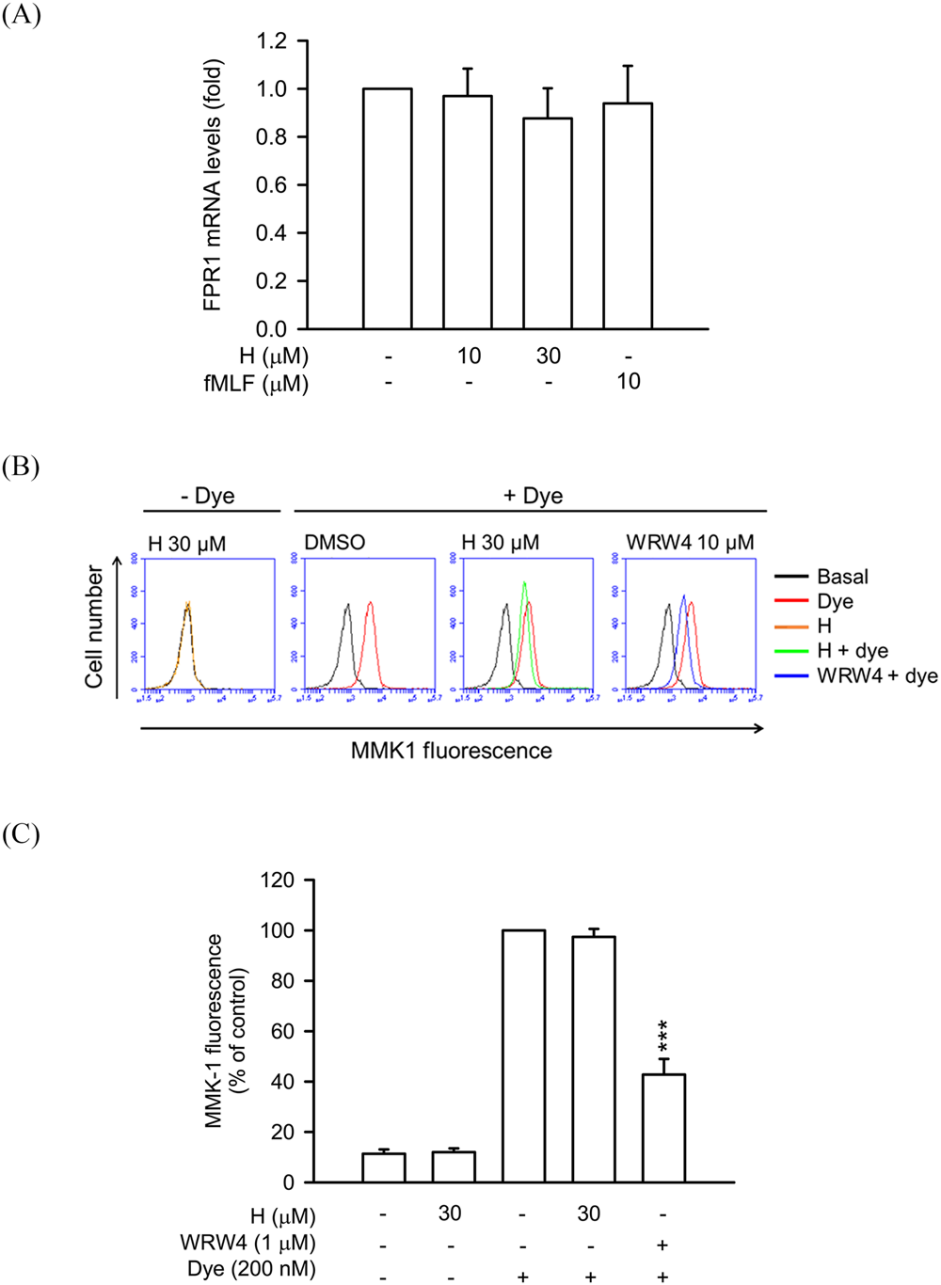
Honokiol does not alter FPR1 mRNA expression and FPR2 binding effect in human neutrophils. (**A**) Human neutrophils were treated with 0.1% DMSO (as control), honokiol (10 and 30 μM), or fMLF (10 μM) at 4 °C for 30 min. Total RNAs were isolated with Trizol reagent and FPR1 mRNA levels were analyzed by quantitative PCR. (**B**) Human neutrophils were incubated with 0.1% DMSO (as control), honokiol (H; 10 μM), or WRW4 (1 μM) for 10 min and then labelled with FPR2-specific fluorescent ligand MMK-1F (200 nM) for 15 min. Representative histograms showing typical fluorescence in the absence or presence of MMK-1F with honokiol or WRW4. (**C**) Mean fluorescence intensity of (**B**) is shown. All data are expressed as the mean ± S.E.M. (*n* = 3 or 4). ***P < 0.001 compared with fluorescent dye alone.

To further investigate the specific binding activity of honokiol in human neutrophils, the expression of FPR1 mRNA and receptor binding effect of FPR2 were studied. As shown in Fig. 6A, treatment of honokiol and fMLF for 30 min did not alter the FPR1 mRNA levels in human neutrophils, ruling out the effect of honokiol on mRNA level. Human FPR family has three members, FPR1, FPR2, and FPR3. Human neutrophils contain FPR1 and FPR2^23, 30^. The receptor binding effect of Leu-Glu-Ser-Ile-Phe-Arg-Ser-Leu-Leu-Phe-Arg-Val-Met-fluorescein (MMK-1F), an FPR2-specific fluorescent ligand, on the surface of neutrophils was evaluated. The results showed that WRW4 (a FPR2 antagonist), but not honokiol, inhibited the binding of MMK-1F on neutrophils (Fig. 6B and C).

### Honokiol inhibits dose–response effects of fMLF in human neutrophils

**Figure 7:**
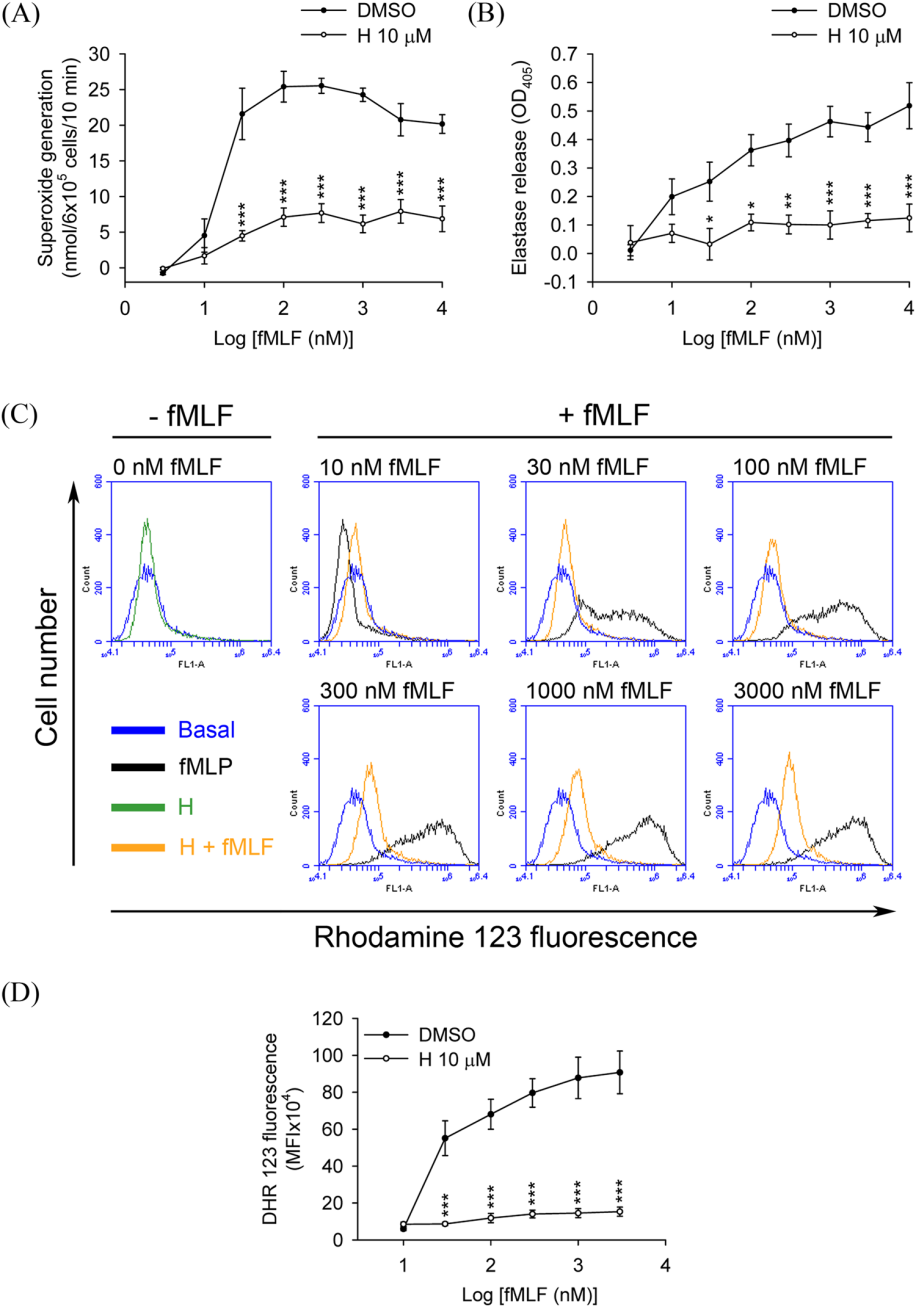
Honokiol inhibits dose–response effects of fMLF in human neutrophils. Human neutrophils were incubated with 0.1% DMSO (as control) or honokiol (H; 10 μM) for 5 min before the addition of different concentrations of fMLF in the presence of CB. (**A**) Superoxide anion production was measured by assessing ferricytochrome *c* reduction. (**B**) Elastase release was measured spectrophotometrically at 405 nm. (**C**) Representative histograms of rhodamine 123 are shown. (**D**) Mean fluorescence intensities of (**C**) are shown. All data are expressed as the mean ± S.E.M. (*n* = 3–5). *P < 0.05; **P < 0.01; ***P < 0.001 compared with corresponding control.

**Figure 8:**
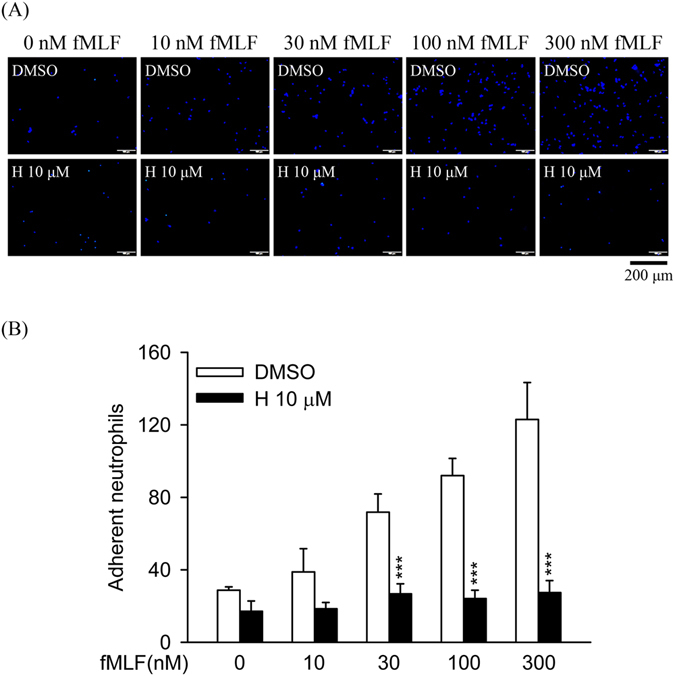
Honokiol inhibits fMLF-induced adhesion of human neutrophils to endothelial cells (ECs). Hoechst-labelled neutrophils were incubated with 0.1% DMSO (as control) or honokiol (H; 10 μM) for 5 min before activation with different concentrations of fMLF and priming with CB. Activated neutrophils were then co-cultured with LPS-pre-activated ECs for 15 min. (**A**) Adherent neutrophils on ECs were detected using microscopy. Bar (bottom), 200 μm. (**B**) Adherent neutrophils were measured and are shown as the mean ± S.E.M. (*n* = 6). **P < 0.01 compared with control.

The dose–response effects of fMLF (10–3000 nM) on superoxide anion generation, elastase release, and ROS formation were inhibited in the presence of honokiol (10 μM). The maximal effects of fMLF on these responses were significantly suppressed by honokiol (Fig. 7). These results also demonstrated that honokiol inhibited FPR1-mediated neutrophil respiratory burst and degranulation through high-affinity manners. The adhesion of neutrophils to tissue is a critical event in the FPR1-induced inflammatory response. In line with these results, honokiol significantly reduced fMLF-induced human neutrophils adherent to cerebral ECs (Fig. 8).

### Honokiol exerts inhibitory effects on calcium (Ca^2+^) mobilization and MAPKs activation in fMLF-activated human neutrophils

**Figure 9:**
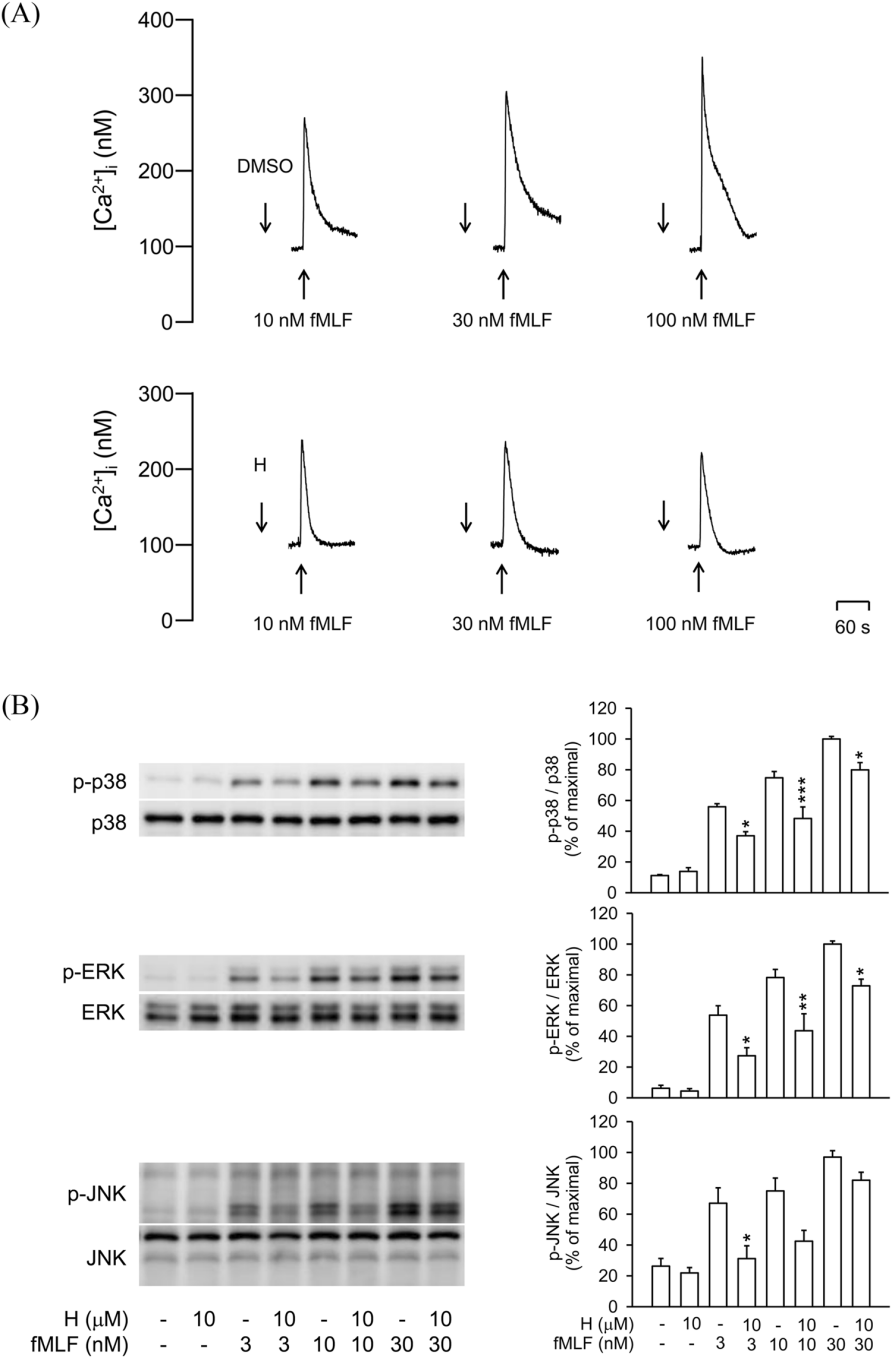
Honokiol inhibits Ca^2+^ mobilization and MAPKs phosphorylation in fMLF-activated human neutrophils. (**A**) Fluo-3/AM-labelled human neutrophils were incubated with 0.1% DMSO (as control) or honokiol (H; 10 μM) for 5 min. Cells were activated by fMLF and mobilization of Ca^2+^ was determined in real time by using a spectrofluorometer. Representative traces are shown (*n* = 7). (**B**) Human neutrophils were incubated with 0.1% DMSO (as the control) or honokiol (10 μM) for 5 min before stimulation with or without fMLF for another 25 s. Phosphorylation of p38, ERK, and JNK was analyzed by immunoblotting with antibodies against the phosphorylated and total form of each protein. All the Western blotting experiments were performed under the same condition. After transferred the blots onto nitrocellulose membranes, we immediately cropped the targeted blots according to referenced indicating markers, and then targeted proteins were immunoblotted with its specific monoclonal antibody. Data are normalized to the corresponding total protein level and expressed as mean ± S.E.M. relative to the mean maximal ratio (*n* = 4). *P < 0.05; **P < 0.01; ***P < 0.001 compared with corresponding control.

Intracellular Ca^2+^ and MAPKs play important roles in several neutrophil functions. To determine whether treatment with honokiol alters Ca^2+^ mobilization and MAPKs activation in fMLF-activated human neutrophils, intracellular Ca^2+^ concentration ([Ca^2+^]_i_) and phosphorylation of MAPKs were assayed. Treatment with fMLF led to a rapid and transient increase in [Ca^2+^]_i_ in human neutrophils. Peak [Ca^2+^]_i_ caused by fMLF was significantly diminished by honokiol (10 μM) (Fig. 9A). In addition, stimulation of human neutrophils with fMLF caused rapid phosphorylation of p38 MAPK, ERK, and JNK in dose-dependent manners. Similarly, honokiol showed inhibitory effects on the phosphorylation of p38 MAPK, ERK, and JNK (Fig. 9B).

### Honokiol reduces fMLF-stimulated neutrophils accumulation in the peritoneal cavity in mice

**Figure 10:**
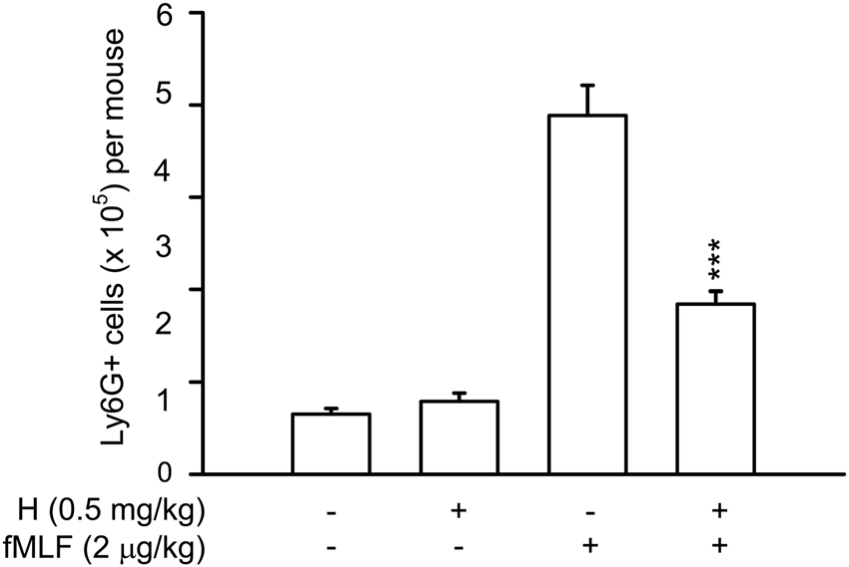
Honokiol inhibits neutrophils accumulation in the peritoneal cavity in fMLF-stimulated mice. Male BALB/c mice aged 7–8 weeks were pretreated by intraperitoneal injection of honokiol (0.5 mg/kg body weight) or vehicle alone (10% DMSO/normal saline) for 30 min, and then challenged with intraperitoneal injection of fMLF (2 μg/kg in normal saline) for 2 h. The peritoneal cells were harvested and stained with the Ly6G (Gr-1) rat anti-mouse, PE conjugated monoclonal antibody for 30 min at 4 °C. The number of Ly6G positive cells were detected by flow cytometry. Data are expressed as the mean ± S.E.M. (*n* = 7 in each group). ***P < 0.001 compared with fMLF.

To test whether honokiol has anti-inflammatory activity *in vivo*, fMLF-induced neutrophils accumulation in the peritoneal cavity of BALB/c mice were studied. Intraperitoneal injection of fMLF for 2 h greatly induced accumulation of Ly6G positive neutrophils in the peritoneal cavity of mice. fMLF-induced neutrophils recruitment into the peritoneal cavity in mice was significantly suppressed by pre-intraperitoneal injection of honokiol (Fig. 10). This result showed that *in vivo* chemotaxis of mouse neutrophils induced by FPR1 was also suppressed by honokiol.

## Discussion

Honokiol is an important bioactive component of *Magnolia officinalis*, which has been utilized as an herbal medicine for many years in Asian countries. Previous studies have shown that honokiol has potent anti-inflammatory effects^11, 14–18, 20^. Human neutrophils play important roles in the pathogenesis of various injuries and diseases, including traumatic shock, ischemia-reperfusion injury, acute coronary artery disease, respiratory distress, and sepsis^31–34^. Respiratory burst and degranulation in activated neutrophils have noteworthy effects in these inflammatory diseases^4, 5, 35^. Several potentially anti-microbial and pro-inflammatory proteases can be found in neutrophil granules. Elastase is an abundant serine protease secreted by activated human neutrophils^36, 37^. Superoxide anion and elastase released from activated neutrophils can directly or indirectly cause organ damage by destroying the surrounding tissues. However, the pharmacological mechanisms of honokiol in human neutrophils are not well understood. In the present study, we found that honokiol inhibited respiratory burst, degranulation, and adhesion in fMLF-activated human neutrophils. Further studies suggest that the anti-inflammatory effects of honokiol are mediated through blockade of FPR1.

FPRs are expressed on various immune cells and play a significant role in different inflammatory responses^38^. Two members of this family, FPR1 and FPR2, are expressed on human neutrophils^23, 30^. FPR1 has a high affinity to formyl peptides produced by bacteria or released from damaged mitochondria during infection or tissue injury^25–27^. Our results showed that honokiol dose-dependently blocked FPR1-specific ligand fNLFNYK binding^29^ to FPR1 in human neutrophils, suggesting that honokiol has FPR1 blocking activity. To support this hypothesis, honokiol inhibited fNLFNYK binding to FPR1 in dibutyryl cAMP-differentiated neutrophil-like THP-1 cells and hFPR1-transfected HEK293 cells. These results indicate that honokiol is a FPR1 inhibitor. Moreover, two FPR1 agonists, fMLF (bacteria formyl peptide) and fMMYALF (mitochondria-derived formyl peptide)^28^, induced superoxide anion generation and elastase release were inhibited by honokiol in human neutrophils in dose-dependent manners.

Previous studies have shown that honokiol has free radical scavenging activity^39, 40^. In line with these findings, our data showed that ROS formation is more sensitive to inhibition by honokiol than superoxide anion generation in fMLF-stimulated human neutrophils. Honokiol has been shown to scavenge superoxide anion in melanoma cells^40^ and to inhibit PMA or fMLF induced ROS production in rat neutrophils^15, 41^. In this study, honokiol at concentration up to 10 μM did not significantly suppress superoxide anion generation in PMA-activated human neutrophils and in a cell-free xanthine/xanthine oxidase assay. These conflicting results may be attributed to differences in experimental conditions, ROS assays, and animal species.

Honokiol showed significant inhibitory effects on FPR1-mediated superoxide anion production, ROS formation, elastase release, and cell adhesion in human neutrophils, confirming that honokiol has potential to reduce the harmful effects of neutrophils. Additionally, higher concentrations of fMLF induced cell responses were considerably suppressed by honokiol, indicating that honokiol binds to FPR1 in a high-affinity manner. This conclusion is supported further by receptor-binding assay showing that FPR1 binding effect of fNLFNYK, even at high concentrations, were still inhibited by honokiol.

The intracellular signalling pathways of neutrophil activation are complex. FPR1 is a Gi protein–coupled receptor, and its activation can trigger phospholipase C to convert phosphoinositol 4,5-biphosphate to inositol 1,4,5-triphosphate, and subsequently stimulate the rapid release of intracellular Ca^2+^ ^42, 43^. The second messengers, ERK, p38 MAPK and JNK, are also known to play significant roles in regulation of various neutrophil immune responses after FPR1 activation^44–46^. FPR1 antagonists exert anti-inflammatory and tissue-protective effects. Previous reports showed that FPR1 antagonists attenuate fMLF-stimulated Ca^2+^ influx and MAPKs phosphorylation by blocking FPR1^47–50^. In the present study, honokiol showed significant inhibitory effects on Ca^2+^ influx as well as phosphorylation of ERK, p38 MAPK and JNK in fMLF-induced human neutrophils. These results demonstrate that honokiol controls neutrophil activations by attenuating the downstream signaling pathways of FPR1. In summary, our studies provide evidence that honokiol can act as FPR1 inhibitor that reduces *N*-formyl peptides-induced neutrophil respiratory burst, degranulation, chemotaxis, and adhesion. We therefore suggest that honokiol has therapeutic potential as an adjunct for the treatment of FPR1-mediated neutrophilic inflammatory diseases.

## Methods

### Reagents

Honokiol was isolated from *Magnolia* cortex according to our previous report^51^ and dissolved in DMSO. Human *N*-formyl peptide [fMMYALF, the *N*-terminal sequence of mitochondrial NADH dehydrogenase subunit 6 (ND6)] was synthesized by GeneDireX (NV, USA). Hank’s balanced salt solution (HBSS) was obtained from GIBCO (Grand Island, NY, USA). CytoTox 96 Non-Radioactive Cytotoxicity assay kits were obtained from Promega (Madison, Wisconsin, USA). fNLFNYK, fluo-3/AM, and dihydrorhodamine 123 (DHR 123) were purchased from Molecular Probes (Eugene, OR, USA) and 2-(4-iodophenyl)-3-(4-nitrophenyl)-5-(2,4-disulfophenyl)-2H-tetrazolium monosodium salt (WST-1) was purchased from Dojindo (Kurnamoto, Japan). The antibodies against MAPKs were obtained from Cell Signaling (Beverly, MA, USA). Other pharmacologic agents were purchased from Sigma-Aldrich (St. Louis, MO, USA).

### Isolation of human neutrophils

This study was reviewed and approved by the Institutional Review Board of Chang Gung Memorial Hospital, and written informed consent was obtained from each volunteer. The methods were performed in accordance with the approved guidelines. Blood was collected from healthy volunteers (aged 20–32 years). Neutrophils were isolated from peripheral blood according to the standard dextran sedimentation procedure followed by centrifugation in a Ficoll-Hypaque gradient and hypotonic lysis of erythrocytes. The purified neutrophils contained approximately 98% viable cells, as determined using trypan blue exclusion, and were suspended in calcium-free HBSS at 4 °C before use^52^.

### Preparation of human monocytic leukaemia (THP-1) cells

The THP-1 human leukaemia monocytic cell line was cultured in RPMI 1640 medium supplemented with fetal bovine serum (FBS; Biological Industries, Israel), glutamine (2 mM), and antibiotics (100 μg/ml streptomycin, 0.25 μg/ml amphotericin B and 100 U/ml penicillin). THP-1 cell differentiation was performed by culturing cells in 300 μM dibutyryl cAMP for 48 h^49^.

### Preparation of brain ECs

Mice brain microvascular ECs (bEnd.3) were obtained from the Bioresource Collection Center (Hsinchu, Taiwan). Cultured cells were maintained in a humidified atmosphere (37 °C, 5% CO2) with Dulbecco’s modified Eagle’s medium (DMEM, Gibco, Grand Island, NY) supplemented with 10% FBS and antibiotics. ECs were grown to confluence and passaged every 3 days at 1 × 10^5^ cells/ml^18^.

### Expression of FPR1 in human embryonic kidney cells (HEK-293)

HEK-293 cells were maintained in DMEM supplemented with 10% FBS, 2 mM glutamine, and antibiotics. HEK-293 were stably transfected with the pCMV6-AC vector containing the human FPR1 gene (NM_002029; OriGene, Rockville, MD) using X-treme GENE Hp DNA transfection reagent (Roche, Mannheim, Germany). FPR1-expressed HEK-293 were cultured in the medium containing G418 (2 mg/ml) for further studies^48^.

### Measurement of superoxide anion generation

The SOD-inhibitable reduction of ferricytochrome *c* was used to measure superoxide anion release from human neutrophils. Neutrophils (6 × 10^5^ cells/ml) were pre-incubated with 0.5 mg/ml ferricytochrome *c* and 1 mM CaCl_2_ at 37 °C for 5 min, and then treated with 0.1% DMSO (as control) or honokiol (1, 3, or 10 μM) for 5 min before cell activation. Neutrophils were activated with fMLF, fMMYALF, or PMA for a further 10 min. When fMLF and fMMYALF were used as activators, cells were primed by pre-incubation for 3 min with cytochalasin B (CB, 1 μg/ml). The change in absorbance at 550 nm reflected the reduction in ferricytochrome *c* and was monitored continuously by using a spectrophotometer (U-3010; Hitachi, Tokyo, Japan). Superoxide anion production was calculated as described previously^52^.

### Analysis of elastase release

Elastase release was evaluated as degranulation in activated neutrophils. Neutrophils (6 × 10^5^ cells/ml) were pre-incubated with methoxysuccinyl-Ala-Ala-Pro-Val-p-nitroanilide (100 μM) at 37 °C and honokiol (1, 3, or 10 μM) was then added for 5 min before treatment with fMLF or fMMYALF for a further 10 min with CB (0.5 μg/ml) priming. The change in absorbance at 405 nm was analysed continuously with a spectrophotometer^53^.

### Measurement of ROS formation

ROS production was determined from the conversion of non-fluorescent DHR 123 to fluorescent rhodamine 123, detected using flow cytometry. Neutrophils (2 × 10^6^ cells/ml) were incubated with DHR 123 (2 μM) for 15 min at 37 °C, and then treated with honokiol (0.1–10 μM) for 5 min before the addition of fMLF/CB (0.5 μg/ml) for a further 5 min. The change in fluorescence was analysed using an Accuri C6 flow cytometer (BD Biosciences, CA, USA)^49^.

### Cytotoxicity

Cytotoxicity was determined using LDH assay kits (Promega, Madison, WI, USA). Neutrophils (5 × 10^6^ cells/ml) were treated with 0.1% DMSO (as control) or honokiol (1, 3, and 10 μM) for 15 min at 37 °C. Cell-free supernatants were collected and LDH was measured according to the manufacturer’s instructions. Total LDH activity was determined following the lysis of neutrophils using 0.1% Triton X-100 at 37 °C^49^.

### Superoxide anion-scavenging assay

The superoxide anion-scavenging effect of honokiol was measured in a cell-free xanthine/xanthine oxidase system. Assay buffer [Tris (pH 7.4), 0.02 U/ml XO, and 0.3 mM WST-1] was pre-incubated with 0.1% DMSO (as control), honokiol (1, 3, and 10 μM), or SOD (20 U/ml, as positive control) for 3 min prior to the addition of 0.1 mM xanthine for another 10 min at 30 °C. The change in absorbance, indicating the reduction of WST-1 by superoxide anion, was determined at 450 nm^52^.

### Receptor-binding assay

fNLFNYK (a fluorescent analogue of fMLF) or MMK-1F (a fluorescent analogue of MMK-1) was used for receptor binding assays^49^. Neutrophils, differentiated THP-1, or FPR1-expressed HEK-293 were pre-incubated with honokiol, fMLF, or WRW4 for 10 min at 4 °C and then labelled with fNLFNYK for 20 min or MMK-1F for 15 min. Cells were immediately assayed by flow cytometry.

### Neutrophil adhesion

Hoechst 33342 (1 ng/ml, Invitrogen)-labelled neutrophils were treated with 0.1% DMSO or honokiol (10 μM) for 5 min prior to treatment with fMLF (10–300 nM)/CB (1 μg/ml) for another 15 min. ECs were pre-treated with lipopolysaccharide (2 μg/ml) for 4 h. After washing, labelled neutrophils (1 × 10^5^ cells/ml) were incubated with ECs for 15 min. Non-adherent neutrophils were removed by gentle washing with RPMI. Adherent neutrophils on ECs were counted in six randomly selected areas (0.572 mm^2^) under a motorized inverted microscope (IX81, Olympus, Japan) with a 10 × objective^18^.

### Measurement of [Ca^2+^]_i_

Fluo-3-labelled cells were pre-incubated with honokiol (10 μM) for 5 min and then activated using different concentrations of fMLF (10, 30, and 100 nM) at 37 °C. The change in [Ca^2+^]_i_ fluorescence was measured using a spectrofluorometer (Hitachi F-4500) under continuous stirring with an excitation wavelength of 488 nm and emission wavelength of 520 nm^53^.

### RNA extraction, cDNA synthesis and quantitative PCR

Human neutrophils were treated with 0.1% DMSO (as control), honokiol (10 and 30 μM) or fMLF (10 μM) at 4 °C for 30 min. Total RNAs were isolated with Trizol reagent (Invitrogen) according to the manufacturer’s protocol. 1 μg RNAs were reverse-transcribed into cDNA using iScript™ cDNA Synthesis Kit (Bio-Rad) and sequentially 1/20 of the cDNAs were analyzed by quantitative PCR with Fast Start Universal SYBR Green Master (Roche). The PCR primers are: FPR1, sense (5′-ACAGTCCAGGAGCAGACAAG-3′) and antisense (5′-CAGCAGATACAGCAGGTGTCC-3′); GAPDH, sense (5′-TGCACCACCAACTGCTTAGC-3′) and antisense (5′-GGCATGGACTGTGGTCATGAG-3′).

### Immunoblotting

Human neutrophils were incubated with 0.1% DMSO (as control) or honokiol (10 μM) at 37 °C for 5 min, and then activated by fMLF (3, 10, and 30 nM)/CB (1 μg/ml) for another 25 s. Sample buffer (62.5 mM pH 6.8 Tris-HCl, 4% sodium dodecyl sulfate (SDS), 5% β-mercaptoethanol, 0.0125% bromophenol blue, and 8.75% glycerol) was added to stop the reaction and the mixtures were heated at 100 °C for 15 min. The whole-cell lysates were used for immunoblotting assays. Phosphorylation of MAPKs was identified by immunoblotting with the corresponding antibody overnight at 4 °C, followed by incubation with horseradish peroxidase-conjugated, secondary anti-rabbit antibody (Cell Signaling Technology, Beverly, MA, USA). The labeled proteins were detected using enhanced chemiluminescence kits, and analyzed using a densitometer (UVP, Upland, CA, USA). The quantitative ratios for all samples were normalized to the corresponding total protein^48^.

### fMLF-induced neutrophils recruitment in mouse peritoneal cavity

All animal experiments were performed in accordance with the guidelines of the Animal Welfare Act and The Guide for Care and Use of Laboratory Animals from the National Institutes of Health. Experimental protocols were approved by the Institutional Animal Care and Use Committee of Chang Gung University.

Male BALB/c mice aged 7–8 weeks were purchased from the BioLASCO (Taipei, Taiwan) and housed in an air-conditioned room at 25 °C with a 12 h light and dark cycle. Mice were pretreated by intraperitoneal injection of 100 μl honokiol (0.5 mg/kg body weight) or vehicle alone (10% DMSO/normal saline). After 30 min, the mice were challenged with intraperitoneal injection of fMLF (2 μg/kg in normal saline). Mice were sacrificed at 2 h after fMLF stimulation and the peritoneal cavities were washed with 1 ml of normal saline containing 10 U/ml heparin. The harvested peritoneal cells were mixed with the Ly6G (Gr-1) rat anti-mouse, PE conjugated monoclonal antibody (eBioscience, CA, USA) for 30 min at 4 °C. The number of Ly6G positive cells were detected by flow cytometry^54^.

### Statistical analysis

Results are presented as means ± S.E.M. Statistical analysis was performed with Sigma Plot (Systat Software, San Jose, CA, USA) by using Student’s *t*-test or one-way ANOVA followed by post hoc test for multiple comparisons. A value of *P* < 0.05 was considered statistically significant.

## Electronic supplementary material

Supplementary Information

## References

[1] Kolaczkowska E, Kubes P (2013). Neutrophil recruitment and function in health and inflammation. Nat Rev Immunol.

[2] von Andrian UH (1993). *In vivo* behavior of neutrophils from two patients with distinct inherited leukocyte adhesion deficiency syndromes. J Clin Invest.

[3] Angelillo-Scherrer A (2012). Leukocyte-derived microparticles in vascular homeostasis. Circ Res.

[4] Caielli S, Banchereau J, Pascual V (2012). Neutrophils come of age in chronic inflammation. Curr Opin Immunol.

[5] Amulic B, Cazalet C, Hayes GL, Metzler KD, Zychlinsky A (2012). Neutrophil function: from mechanisms to disease. Annu Rev Immunol.

[6] Bruijnzeel PL, Uddin M, Koenderman L (2015). Targeting neutrophilic inflammation in severe neutrophilic asthma: can we target the disease-relevant neutrophil phenotype?. J Leukoc Biol.

[7] Lam DC (2015). S-maltoheptaose targets syndecan-bound effectors to reduce smoking-related neutrophilic inflammation. Sci Rep.

[8] Tsai, Y. F., Yang, S. C. & Hwang, T. L. Formyl peptide receptor modulators: a patent review and potential applications for inflammatory diseases (2012–2015). *Expert Opin Ther Pat*, 1–18, doi:10.1080/13543776.2016.1216546 (2016). 10.1080/13543776.2016.1216546 27454150

[9] Lin CJ (2016). Preclinical effects of honokiol on treating glioblastoma multiforme via G1 phase arrest and cell apoptosis. Phytomedicine.

[10] Song, J. M. *et al*. Honokiol suppresses lung tumorigenesis by targeting EGFR and its downstream effectors. *Oncotarget*, doi:10.18632/oncotarget.10759 (2016). 10.18632/oncotarget.10759 PMC5295387 27458163

[11] Sulakhiya K (2014). Honokiol abrogates lipopolysaccharide-induced depressive like behavior by impeding neuroinflammation and oxido-nitrosative stress in mice. Eur J Pharmacol.

[12] Kim SY, Kim J, Jeong SI, Jahng KY, Yu KY (2015). Antimicrobial Effects and Resistant Regulation of Magnolol and Honokiol on Methicillin-Resistant Staphylococcus aureus. Biomed Res Int.

[13] Liao CY (2015). CLL2-1, a chemical derivative of orchid 1,4-phenanthrenequinones, inhibits human platelet aggregation through thiol modification of calcium-diacylglycerol guanine nucleotide exchange factor-I (CalDAG-GEFI). Free Radic Biol Med.

[14] Wang Y (2013). Honokiol protects rat hearts against myocardial ischemia reperfusion injury by reducing oxidative stress and inflammation. Exp Ther Med.

[15] Liou KT, Shen YC, Chen CF, Tsao CM, Tsai SK (2003). Honokiol protects rat brain from focal cerebral ischemia-reperfusion injury by inhibiting neutrophil infiltration and reactive oxygen species production. Brain Res.

[16] Liu J (2015). Honokiol downregulates Kruppel-like factor 4 expression, attenuates inflammation, and reduces histopathology after spinal cord injury in rats. Spine (Phila Pa 1976).

[17] Zhu X, Wang Z, Hu C, Li Z, Hu J (2014). Honokiol suppresses TNF-alpha-induced migration and matrix metalloproteinase expression by blocking NF-kappaB activation via the ERK signaling pathway in rat aortic smooth muscle cells. Acta Histochem.

[18] Chen PJ (2016). Honokiol suppresses TNF-alpha-induced neutrophil adhesion on cerebral endothelial cells by disrupting polyubiquitination and degradation of IkappaBalpha. Sci Rep.

[19] Pende A, Artom N, Bertolotto M, Montecucco F, Dallegri F (2016). Role of neutrophils in atherogenesis: an update. Eur J Clin Invest.

[20] Wang XD, Wang YL, Gao WF (2015). Honokiol possesses potential anti-inflammatory effects on rheumatoid arthritis and GM-CSF can be a target for its treatment. Int J Clin Exp Pathol.

[21] Fu H (2006). Ligand recognition and activation of formyl peptide receptors in neutrophils. J Leukoc Biol.

[22] Zhang Y (2003). Evaluation of human leukocyte N-formylpeptide receptor (FPR1) SNPs in aggressive periodontitis patients. Genes Immun.

[23] Le Y, Murphy PM, Wang JM (2002). Formyl-peptide receptors revisited. Trends Immunol.

[24] Dorward DA (2015). The role of formylated peptides and formyl peptide receptor 1 in governing neutrophil function during acute inflammation. Am J Pathol.

[25] Carp H (1982). Mitochondrial N-formylmethionyl proteins as chemoattractants for neutrophils. J Exp Med.

[26] Wenceslau CF, McCarthy CG, Szasz T, Goulopoulou S, Webb RC (2015). Mitochondrial N-formyl peptides induce cardiovascular collapse and sepsis-like syndrome. Am J Physiol Heart Circ Physiol.

[27] Yang SC, Hwang TL (2016). The potential impacts of formyl peptide receptor 1 in inflammatory diseases. Front Biosci (Elite Ed).

[28] Rabiet MJ, Huet E, Boulay F (2005). Human mitochondria-derived N-formylated peptides are novel agonists equally active on FPR and FPRL1, while Listeria monocytogenes-derived peptides preferentially activate FPR. Eur J Immunol.

[29] Ye RD, Cavanagh SL, Quehenberger O, Prossnitz ER, Cochrane CG (1992). Isolation of a cDNA that encodes a novel granulocyte N-formyl peptide receptor. Biochem Biophys Res Commun.

[30] Ye RD (2009). International Union of Basic and Clinical Pharmacology. LXXIII. Nomenclature for the formyl peptide receptor (FPR) family. Pharmacol Rev.

[31] Liao Y, Liu P, Guo F, Zhang ZY, Zhang Z (2013). Oxidative burst of circulating neutrophils following traumatic brain injury in human. PLoS One.

[32] Schofield ZV, Woodruff TM, Halai R, Wu MC, Cooper MA (2013). Neutrophils–a key component of ischemia-reperfusion injury. Shock.

[33] Sahinarslan A (2011). Plasma neutrophil gelatinase-associated lipocalin levels in acute myocardial infarction and stable coronary artery disease. Coron Artery Dis.

[34] Iwata K (2010). Effect of neutrophil elastase inhibitor (sivelestat sodium) in the treatment of acute lung injury (ALI) and acute respiratory distress syndrome (ARDS): a systematic review and meta-analysis. Intern Med.

[35] Zhang W, Voice J, Lachmann PJ (1995). A systematic study of neutrophil degranulation and respiratory burst *in vitro* by defined immune complexes. Clin Exp Immunol.

[36] Muley MM (2016). Neutrophil elastase induces inflammation and pain in mouse knee joints via activation of proteinase-activated receptor-2. Br J Pharmacol.

[37] Tsai YF, Hwang TL (2015). Neutrophil elastase inhibitors: a patent review and potential applications for inflammatory lung diseases (2010–2014). Expert Opin Ther Pat.

[38] Li L (2016). New development in studies of formyl-peptide receptors: critical roles in host defense. J Leukoc Biol.

[39] Murakami Y (2012). Comparative inhibitory effects of magnolol, honokiol, eugenol and bis-eugenol on cyclooxygenase-2 expression and nuclear factor-kappa B activation in RAW264.7 macrophage-like cells stimulated with fimbriae of Porphyromonas gingivalis. In Vivo.

[40] Dikalov S, Losik T, Arbiser JL (2008). Honokiol is a potent scavenger of superoxide and peroxyl radicals. Biochem Pharmacol.

[41] Liou KT, Shen YC, Chen CF, Tsao CM, Tsai SK (2003). The anti-inflammatory effect of honokiol on neutrophils: mechanisms in the inhibition of reactive oxygen species production. Eur J Pharmacol.

[42] Migeotte I, Communi D, Parmentier M (2006). Formyl peptide receptors: a promiscuous subfamily of G protein-coupled receptors controlling immune responses. Cytokine Growth Factor Rev.

[43] Lavigne MC, Murphy PM, Leto TL, Gao JL (2002). The N-formylpeptide receptor (FPR) and a second G(i)-coupled receptor mediate fMet-Leu-Phe-stimulated activation of NADPH oxidase in murine neutrophils. Cell Immunol.

[44] Hwang TL (2010). The hederagenin saponin SMG-1 is a natural FMLP receptor inhibitor that suppresses human neutrophil activation. Biochem Pharmacol.

[45] Mocsai, A. *et al*. Kinase pathways in chemoattractant-induced degranulation of neutrophils: the role of p38 mitogen-activated protein kinase activated by Src family kinases. *J Immunol* **164**, 4321–4331, doi:ji_v164n8p4321 [pii] (2000). 10.4049/jimmunol.164.8.4321 10754332

[46] Dang PM (2006). A specific p47phox -serine phosphorylated by convergent MAPKs mediates neutrophil NADPH oxidase priming at inflammatory sites. J Clin Invest.

[47] Hannigan M (2002). Neutrophils lacking phosphoinositide 3-kinase gamma show loss of directionality during N-formyl-Met-Leu-Phe-induced chemotaxis. Proc Natl Acad Sci USA.

[48] Yang SC (2013). Bioactive secondary metabolites of a marine Bacillus sp. inhibit superoxide generation and elastase release in human neutrophils by blocking formyl peptide receptor 1. Molecules.

[49] Yang SC (2013). Propofol inhibits superoxide production, elastase release, and chemotaxis in formyl peptide-activated human neutrophils by blocking formyl peptide receptor 1. J Immunol.

[50] Yang SC (2017). Dipeptide HCH6-1 inhibits neutrophil activation and protects against acute lung injury by blocking FPR1. Free Radic Biol Med.

[51] Lin CF (2013). Maximizing dermal targeting and minimizing transdermal penetration by magnolol/honokiol methoxylation. Int J Pharm.

[52] Hwang TL (2003). Soluble guanylyl cyclase activator YC-1 inhibits human neutrophil functions through a cGMP-independent but cAMP-dependent pathway. Mol Pharmacol.

[53] Hwang TL (2009). Suppression of superoxide anion and elastase release by C18 unsaturated fatty acids in human neutrophils. J Lipid Res.

[54] Polesskaya O (2014). MLK3 regulates fMLP-stimulated neutrophil motility. Mol Immunol.

