# Supplementary material for: Honokiol suppresses formyl peptide-induced human neutrophil activation by blocking formyl peptide receptor 1
Source: Sci Rep. 2017 Jul 27;7:6718. doi: 10.1038/s41598-017-07131-w (PMC5532207; doi:10.1038/s41598-017-07131-w)
Supplement: Supplementary file 1 — Supplementary Information [file 41598_2017_7131_MOESM1_ESM.pdf]

Honokiol suppresses formyl peptide-induced human neutrophil activation by blocking formyl peptide receptor 1

Fu-Chao Liu, Huang-Ping Yu, Yu-Ting Syu, Jia-You Fang, Chwan-Fwu Lin, Shih-Hsin Chang , Yen-Tung Lee, Tsong-Long Hwang

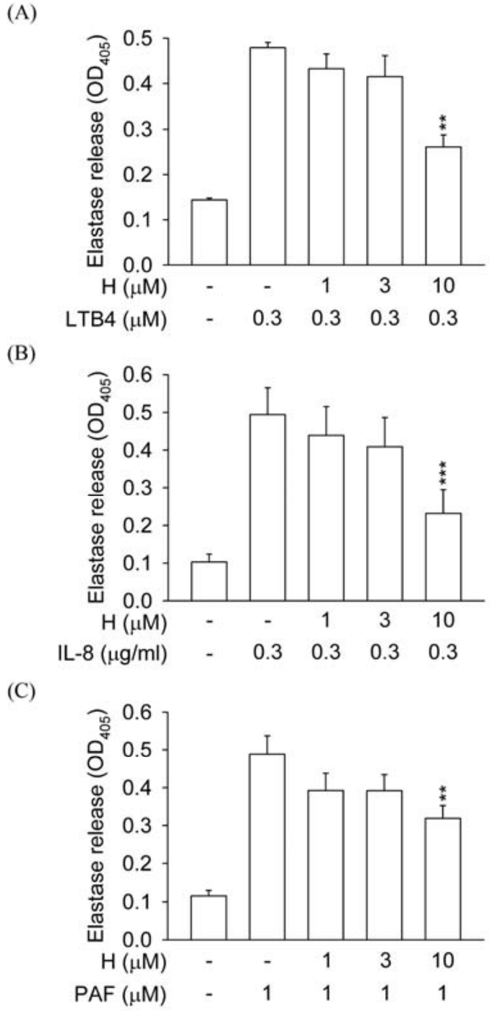

Fig. S1. Effects of honokiol on elastase release in chemoattractants-activated human neutrophils. Human neutrophils were incubated with 0.1% DMSO (as control) or honokiol (H; 1, 3, and 10 μM) for 5 min, and then activated by (A) LTB4 (0.3 μM)/CB (0.5 μg/ml), (B) IL-8 (0.3 μg/ml)/CB (2 μg/ml), and (C) PAF (1 μM)/CB (0.5 μg/ml)f or another 10 min. Elastase release was measured spectrophotometrically at 405 nm. All data are expressed as the mean ± S.E.M. (*n* = 3 or 4). ** $P < 0.01$, *** $P < 0.001$ compared with respective chemoattractant alone.
